# Prevention of severe infectious complications after colorectal surgery using preoperative orally administered antibiotic prophylaxis (PreCaution): study protocol for a randomized controlled trial

**DOI:** 10.1186/s13063-018-2439-4

**Published:** 2018-01-19

**Authors:** Tessa Mulder, Marjolein F. Q. Kluytmans-van den Bergh, Anne Marie G. A. de Smet, Nils E. van ‘t Veer, Daphne Roos, Stavros Nikolakopoulos, Marc J. M. Bonten, Jan A. J. W. Kluytmans, PreCaution Study Group, J. A. J. W. Kluytmans, T. Mulder, M. F. Q. Kluytmans-van den Bergh, F. Kloosterman, M. J. M. Bonten, S. Nikolakopoulos, N. E. van ’t Veer, R. M. P. H. Crolla, G. P. van der Schelling, R. J. de Vos tot Nederveen Cappel, J. Veenemans, A. R. M. Brandt, M. Vos, P. M. Verheijen, A. J. L. Weersink, D. Roos, E. van der Vorm, A. Smits, B. Vlaminckx, E. Bathoorn, B. van Etten, A. M. G. A. de Smet

**Affiliations:** 1 Division Julius Center for Health Sciences and Primary Care, University Medical Center Utrecht, Utrecht University, Utrecht, The Netherlands; 2 Amphia Academy Infectious Disease Foundation, Amphia Hospital, Breda, The Netherlands; 3 Laboratory for Microbiology and Infection Control, Amphia Hospital, Breda, The Netherlands; 4 Department of Critical Care, University Medical Center Groningen, University of Groningen, Groningen, The Netherlands; 5 Department of Clinical Pharmacy, Amphia Hospital, Breda, The Netherlands; 6 Department of Surgery, Reinier de Graaf Gasthuis, Delft, The Netherlands; 7 Department of Medical Microbiology, University Medical Center Utrecht, Utrecht University, Utrecht, The Netherlands

**Keywords:** Surgical site infection, Colorectal surgery, Preoperative oral antibiotic prophylaxis, Infection control, Randomized controlled trial, Placebo, Drug intervention trial, Mortality

## Abstract

**Background:**

Colorectal surgery is frequently complicated by surgical site infections (SSIs). The most important consequences of SSIs are prolonged hospitalization, an increased risk of surgical reintervention and an increase in mortality. Perioperative intravenously administered antibiotic prophylaxis is the standard of care to reduce the risk of SSIs. In the last few decades, preoperative orally administered antibiotics have been suggested as additional prophylaxis to further reduce the risk of infection, but are currently not part of routine practice in most hospitals. The objective of this study is to evaluate the efficacy of a preoperative orally administered antibiotic prophylaxis (Pre-OP) in addition to intravenously administered perioperative antibiotic prophylaxis to reduce the incidence of deep SSIs and/or mortality after elective colorectal surgery.

**Methods/design:**

The PreCaution trial is designed as a multicenter, double-blind, randomized, placebo-controlled clinical trial that will be carried out in The Netherlands. Adult patients who are scheduled for elective colorectal surgery are eligible to participate. In total, 966 patients will be randomized to receive the study medication. This will either be Pre-OP, a solution that consists of tobramycin and colistin sulphate, or a placebo solution. The study medication will be administered four times daily during the 3 days prior to surgery. Perioperative intravenously administered antibiotic prophylaxis will be administered to all patients in accordance with national infection control guidelines. The primary endpoint of the study is the cumulative incidence of deep SSIs and/or mortality within 30 days after surgery. Secondary endpoints include both infectious and non-infectious complications of colorectal surgery, and will be evaluated 30 days and/or 6 months after surgery.

**Discussion:**

To date, conclusive evidence on the added value of preoperative orally administered antibiotic prophylaxis in colorectal surgery is lacking. The PreCaution trial should determine the effects of orally administered antibiotics in preventing infectious complications in elective colorectal surgery.

**Trial registration:**

Netherlands Trial Register, ID: NTR6113. Registered on 11 October 2016; EudraCT 2015-005736-17.

**Electronic supplementary material:**

The online version of this article (doi:10.1186/s13063-018-2439-4) contains supplementary material, which is available to authorized users.

## Background

Surgical site infections (SSIs) are the most common hospital-acquired infections in surgical patients [1, 2]. SSIs are associated with extended hospitalization and are an important source of readmissions and surgical reintervention [2–4]. Relative to other surgical procedures, the incidence of SSIs is highest after colorectal resections, affecting up to 15% of all patients [5]. Despite extensive efforts that have been made in improving infection control practice, the incidence of SSIs after colorectal surgery remains unaffectedly high, whereas the incidence appears to have declined in other surgical specialties [6]. This persisting infection rate highlights the need to explore novel measures to reduce SSIs after colorectal procedures.

To establish new infection prevention measures, it is important to understand the pathogenesis of SSIs. The development of these infections is preceded by microbial contamination of the surgical site [7]. The colon and rectum are densely colonized by microorganisms which explains the high postoperative infection rate [8, 9], as well as the finding that the microorganisms that are most frequently isolated from colorectal SSIs are also the colonizing species [10–12].

An important measure to reduce the risk of postoperative infections is to intravenously administer perioperative prophylactic antibiotics that cover these species, conforming to the national infection control guidelines [13]. For colorectal surgery, orally administered antibiotics can be applied in addition to the intravenously administered prophylaxis. This prophylaxis contains non-absorbable antibiotics, such as neomycin combined with erythromycin or metronidazole, that are administered 1 to 2 days prior to the surgical procedure. The non-absorbable nature of these antibiotics implies almost complete absence of systemic uptake after oral intake. The antibiotics, therefore, only exert local activity in the gastrointestinal tract with low risks of side effects. The rationale of combining systemic and oral prophylaxis is that orally administered antibiotics reduce the colonic bacterial contamination levels directly at the surgical site, whereas systemic antibiotics are used as a safeguard by establishing effective antibiotic concentrations in the soft tissues to minimize the risk of infection and to prevent perioperative endotoxinemia. Despite the beneficial effects of the addition of orally to intravenously administered prophylaxis in preventing infections in individual studies, the oral administration of antibiotics is currently not recommended in international infection control guidelines because of large variability in the antibiotic regimens tested and the limited availability of high-quality studies [14].

The oral administration of antibiotics to reduce the colonic bacterial load became popular as surgical prophylaxis for colorectal surgery in the 1970s [8]. At that time it was believed that orally administered antibiotics would only be effective when the colon was simultaneously cleansed of its contents. Therefore, mechanical bowel preparation (MBP), a technique that involves the administration of osmotic substances to induce voiding of the intestinal contents, was introduced and combined with the preoperative oral administration of antibiotics [9, 15] This combination became standard of care in colorectal surgery. Since then, several studies have questioned the necessity and safety of MBP [15, 16]. Recent meta-analyses have concluded that the use of MBP prior to colorectal surgery could be safely omitted, as MBP alone had no overall beneficial effect on postoperative complications and caused substantial discomfort for the patient [17–21]. Furthermore, MBP has been associated with an increase in inflammatory processes, or with spillage of liquid bowel contents when bowel preparation was performed inadequately resulting in an increase in postoperative infections [16, 22]. Therefore, MBP is no longer recommended in the international guidelines for colorectal surgery. The practice of MBP was also abandoned as the pre-surgical admission period has continuously decreased, precluding these preoperative preparations. The oral administration of antibiotics in an uncleansed colon was thought to be ineffective, resulting in the simultaneous disappearance of oral bowel cleansing and orally administered antibiotic prophylaxis [23].

A recent Dutch single-center, randomized, placebo-controlled trial (RCT) provided new insights into the use of orally administered antibiotics as a surgical prophylaxis prior to gastrointestinal surgery [24]. Instead of using the common regimens for orally administered prophylaxis consisting of tablets, the efficacy of a combination of non-absorbable antibiotics in a suspension was investigated. This suspension contained tobramycin, colistin and amphotericin B and is also used in selective decontamination of the digestive tract (SDD), an infection control measure implemented on intensive care units (ICUs). This study reported a 36% reduction of the incidence of infectious complications and anastomotic leakage in patients receiving the preoperative antibiotic prophylaxis. However, the study included patients who underwent all types of gastrointestinal procedures (*N* = 294). Colorectal procedures were analyzed as a subgroup, resulting in limited statistical power. Furthermore, MBP was frequently administered to patients undergoing colorectal procedures. Nevertheless, the results of this study are promising and encourage further research to confirm the benefit of using orally administered prophylaxis.

In conclusion, the high incidence of SSIs after colorectal surgery and its associated morbidity and mortality justifies the evaluation of new preventive approaches. Preoperative orally administered antibiotic prophylaxis, in addition to standard perioperative intravenously administered prophylaxis, may reduce the incidence of SSIs after colorectal surgery. However, data on its efficacy without the concurrent practice of MBP are lacking [25]. A randomized, double-blind, placebo-controlled clinical trial is, therefore, warranted.

## Methods/design

### Study hypothesis and objectives

The objective of this trial is to determine the efficacy of preoperative orally administered antibiotic prophylaxis (Pre-OP) in addition to perioperative intravenously administered antibiotic prophylaxis on the incidence of deep SSIs and/or mortality after elective colorectal surgery. We hypothesize that Pre-OP will result in a relative reduction of the risk of deep SSIs and/or mortality of at least 40%.

### Study design and setting

The PreCaution study is designed as a double-blind, randomized, placebo-controlled, multicenter trial. The study will be conducted in Dutch university and non-university hospitals. Study centers will be eligible to participate in the study if antibiotic prophylaxis other than the perioperative intravenously administered prophylaxis is not part of the standard perioperative care for colorectal surgery. Patient enrollment started in April of 2017 and is expected to be completed within 18 months after the start of the study.

### Study population

#### Eligibility criteria

Participants will be drawn from a population of adult patients who will undergo elective colorectal surgery. Patients with an absolute contraindication for the study medication, such as pregnant women or nursing mothers, patients with a previously diagnosed allergy for the antibiotics in the study medication or patients with myasthenia gravis, are excluded from participation. Inclusion and exclusion criteria are presented in Table 1.

**Table 1:** Inclusion and exclusion criteria

Inclusion criteria	Exclusion criteria
Adult patients undergoing elective colorectal surgery	Age < 18 years Legally incapacitated patients Patients who are unable to sign informed consent Patients who have an inability to take medication orally Patients who have undergone abdominal surgery within 30 days before randomization Patients with a documented allergy to colistin or aminoglycoside antibiotics Pregnant women or nursing mothers Patients diagnosed with myasthenia gravis Patients with a pre-existent stoma Patients who previously participated in the PreCaution trial or patients who already received study medication but for whom surgery was postponed for more than 7 days

#### Sample size

The sample size is calculated to be 966 patients, based on a one-sided type I error of 2.5%, a type II error of 20% and an assumed 40% reduction on the absolute risk for the primary outcome in the intervention group. The absolute risk of the primary outcome is assumed to be 14%. Furthermore, one interim analysis will be conducted halfway using O’Brien-Fleming type of boundaries, with the possibility of stopping the trial for efficacy in case of overwhelming treatment effect. The absolute risk and the 40% relative risk reduction based on unpublished data from a 4-year survey of a Dutch teaching hospital, where identical preoperative orally administered antibiotic prophylaxis was introduced as standard of care.

#### Evaluable patients

Patients who start with the 3-day intervention period but for whom surgery is cancelled or postponed for more than 7 days, will not be evaluable for analysis of the primary and secondary outcomes. Sample size calculations are based on evaluable patients and, herewith, assumes replacement of all non-evaluable patients. A limit of 7 days is chosen as a cut-off to ensure that patients are not re-colonized at the time of surgery, since it is expected that the effects of decontamination will last for a least several days when the 3-day treatment course is completed.

### Assignment of interventions

#### Randomization

After enrollment, patients will be randomly assigned to one of two treatment arms in a 1:1 ratio.

The randomization will be performed using a permuted block design with varying block sizes and stratification per study center. An independent pharmacist form Stichting Apotheek Haarlemse Ziekenhuizen, who will also be in charge of the production of the study medication, will perform the randomization. The pharmacy will provide the study medication in identical containers that will be sequentially numbered with unique medication numbers. These numbers will correspond to the treatment allocation, and will be documented on the allocation list. This list will be guarded by the hospital pharmacist of the Amphia Hospital (Breda, The Netherlands) who will coordinate the distribution of the study medication to the study centers. Members of the local study teams will be instructed to distribute the study medication according to the allocation sequence. The unique study medication identification numbers will be linked to the subjects at enrollment on a subject identification log.

#### Blinding

Patients, treating physicians and individuals assessing the study outcomes will be blinded to the treatment assignments for the duration of the study. Treatment assignments will be revealed when all patients have completed their 6-month follow-up period.

Individual unblinding will be considered only when the treating physician deems knowledge of the treatment assignment essential for the safety of the patient. Any intentional or unintentional breaking of the blinding will be reported to the sponsor.

### Treatment of subjects

#### Intervention

All patients will receive the study medication as part of the intervention. The intervention can either be treatment with active medication (Pre-OP) or a placebo. Pre-OP is a transparent solution that consists of two antibiotic components: colistin sulphate (20 mg/mL) and tobramycin (16 mg/mL). Placebos are manufactured without the active antibiotic components. The placebo solutions contain flavoring additives and colorants to mimic the taste and color of Pre-OP. All patients will receive perioperative intravenously administered prophylaxis according to the national infection prevention guidelines [26].

#### Dosage and route of administration

Patients are instructed to take the study medication four times daily, during the last 3 days prior to surgery. Each dose consists of 5 mL of either Pre-OP or placebo. In the case of Pre-OP, this represents 100 mg of colistin sulphate and 80 mg of tobramycin.

### Outcome measures

As a primary composite endpoint, we will investigate the efficacy of Pre-OP on deep SSI and/or mortality within 30 days after surgery. The secondary endpoints including their definitions are summarized in Table 2.

**Table 2:** Definition of endpoints

Endpoints	Definitions
Primary composite endpoint	
Cumulative incidence of deep surgical site infection and/or mortality within 30 days after surgery	Deep SSI will be diagnosed according to the CDC criteria for surgical site infections [37]. Deep SSI includes both deep incisional SSI and organ/space SSI
Secondary endpoints	
*30 days after surgery*	
Cumulative incidence of superficial SSI	Superficial and deep SSIs will diagnosed according to the CDC criteria for surgical site infections [37]
Cumulative incidence of deep SSI	
All-cause mortality	
Cumulative incidence of bacteremia	Blood cultures positive for microorganisms
Cumulative incidence of infection with *Clostridium difficile*	Stool sample positive for *C. difficile* toxins
Cumulative incidence of infection with HRE	Clinical cultures positive for ESBL or carbapenemase-producing *Enterobacteriaceae* or *Enterobacteriaceae* resistant to quinolones and/or aminoglycosides [38]
Cumulative incidence of rectal colonization with HRE and colistin resistant species	Rectal swabs positive for HRE or colistin resistant species, measured at baseline and 30 days after surgery
Cumulative incidence of anastomotic leakage	Clinical and/or radiological evidence of leakage requiring surgical or radiological reintervention
Cumulative incidence of re-laparotomy	Reoperation in the abdominal region
In-hospital use of antibiotics	Defined as “days on therapy”
*6 months after surgery*	
All-cause mortality	
Quality of life	Measured with the RAND-36 questionnaire [39]
Length of hospital stay	In days, including all readmissions
Length of ICU stay	In days, including all readmissions
In-hospital costs	

*CDC* Centers for Disease Control and Prevention, *ESBL* extended-spectrum beta-lactamase, *HRE* highly resistant *Enterobacteriaceae*, *ICU* intensive care unit, *SSI* surgical site infection

### Assessment and follow-up

#### Study procedures

The study procedures are listed in Table 3. The preoperative study procedures will take place during visits to the outpatient clinic. These visits will be routinely scheduled and will not be planned as study-specific procedures. During the first visit, the trial will be explained and patients will receive an information letter. Informed consent will be signed during a second preoperative visit. During this visit, the study medication will be provided along with instructions for use and a study diary. Furthermore, the first rectal swab will be taken and patients will receive the first quality-of-life questionnaire. The study diary will be used to document the intake of study medication and to report potential side effects due to the study medication. Patients will take the study medication according to the instructions during the 3 days prior to surgery. The bottles with remainders of study medication will be collected at hospital admission and will be weighted to estimate treatment adherence. The study diaries will also be collected at admission.

**Table 3:** Study flow

	Study period							
	Screening^b^	Enrollment^b^	Intervention			Follow-up and postoperative procedures^c^		
			day					
Time point^a^	~ − 2 weeks	~ − 2 weeks to day − 4	−3	−2	−1	Day 0	Day 30	Month 6
Patient recruitment	X							
Eligibility screening		X						
Informed consent		X						
Allocation		X						
Rectal swab		X					X	
Intake study medication			X	X	X			
Surgery						X		
Estimation of compliance			X	X	X			
Patient characteristics	X	X						
Surgical characteristics						X		
Primary endpoint Deep SSI and/or mortality							X	
Secondary endpoints								
Superficial SSI							X	
Anastomotic leakage							X	
Re-laparotomy							X	
Bacteremia							X	
Rectal colonization with HRE or colistin resistant species							X	
Infection with HRE							X	
Infection with *Clostridium difficile*							X	
In-hospital antibiotic use							X	
Length of hospital stay								X
Length of ICU stay								X
All-cause mortality							X	X
In-hospital costs								X
Quality of life		X						X
Adverse events		X	X	X	X	X	X	X
Serious adverse events		X	X	X	X	X	X	X
SUSARs/SARs			X	X	X	X		

*HRE* highly resistant *Enterobacteriaceae*, *ICU* intensive care unit, *SAR* serious adverse reaction*, SSI* surgical site infection, *SUSAR* serious unexpected adverse reaction

^a^The day of surgery is referred to as day 0. The exact time points of the first and second preoperative visits to the outpatient clinic will depend on the local schedules and procedures

^b^Screening and enrollment are performed during visits to the outpatient clinic. These visits are not study-specific and are planned according to the local logistics

^c^The postoperative data collection is performed by assessment of the patient registers. A phone call will be made at around day 30 and month 6 to assess the status of the patient and to remind the patient to perform the rectal swab (day 30) and fill in the questionnaire (month 6)

Patients will be followed until 6 months after surgery to evaluate the development of primary and secondary outcomes. The data on almost all of these outcomes, including infectious complications will be documented as part of daily practice. Thirty days after surgery, the medical records will be assessed to collect data on primary and secondary outcomes and a second rectal swab will be taken by either the patient or by a member of the local study team. Six months after surgery, the medical records will be assessed again to collect additional data on readmissions and length of hospital stay, including admissions to the ICU. Furthermore, patients will receive the second quality-of-life questionnaire. After the questionnaire has been completed and sent back to the study team, the follow-up is completed.

#### Retention and withdrawal of participants

Patients are free to withdraw from the study at any time, which is in accordance with the Dutch law on medical research on humans. When patients are unwilling to have the rectal swab taken or to fill in the questionnaire, these data will considered to be missing and will be dealt with in data analysis using the appropriate methods.

The intention-to-treat principle will be applied to our primary analysis to deal with poor or non-compliance to the study medication, as this is likely to reflect daily practice. The remainders of study medication will be weighted to estimate the compliance. To enhance the compliance, patients are informed about the bitter taste of the treatment and are advised to take the study medication together with other drinks to mask the taste. Another strategy that will be applied to enhance retention of patients is to call the patients on the two postoperative follow-up moments to remind them about the rectal swab requirement and the questionnaire that will be send to their home address.

#### Microbiological methods

Clinical cultures will be performed and processed in the local microbiology laboratory of the participating centers according to the routine laboratory procedures. Date of culture, specimen type, species cultured, antimicrobial susceptibility and the production of extended-spectrum beta-lactamase or carbapenemase will be documented. Fecal samples from patients who develop nosocomial diarrhea will be tested for the presence of *Clostridium difficile* toxins, according to routine laboratory procedures and based on the indication by the attending physician.

Rectal swabs will be pre-enriched and subsequently cultured using selective media, aimed at the detection of extended-spectrum beta-lactamase (ESBL)-producing *Enterobacteriaceae* and carbapenem-, tobramycin- and colistin-resistant, Gram-negative bacterial species’ identification and antimicrobial susceptibility testing will be performed for all isolates that grow on either one of the screening agars. Phenotypic ESBL confirmation will be performed with the combination disk diffusion method according to the NVMM guideline on the laboratory detection of highly resistant microorganisms (HRMO).

### Data management

An electronic Case Report Form (eCRF) will be used for each patient to collect all data on baseline patient characteristics and outcomes (Additional file 1). The data management department of the Julius Center for Health Sciences and Primary care at the UMC Utrecht developed the eCRF which is hosted by ResearchOnline.

Patients will receive a unique identification number at inclusion. The list with the unique identification numbers linked to the patient will be securely stored at the study site where the patient is included. This list will be the only way to trace the identification numbers back to the individual patients. When the data are entered in the eCRF, patients will be identified with the unique identification number. No patient numbers, names, addresses or complete dates of birth will be recorded.

### Statistical analysis

The primary composite endpoint will be analyzed according to the intention-to-treat principle. All patients who were randomized to receive study medication and who underwent colorectal surgery will be included in this analysis. The difference in the primary outcome between Pre-OP and placebo will be estimated with a Z-test for proportions or by logistic regression, correcting for the stratification variable (study site). Multivariable logistic regression will be used and measured confounders will be fitted in the model as covariates. Secondary outcomes will be analyzed using the chi-square test, Fisher’s exact test, logistic regression, time-to-event analysis, *t* test or Mann–Whitney *U* test, when appropriate. Missing data will be analyzed and handled using.

Exploratory subgroup analyses will be performed for study center, age, sex, Body Mass Index (BMI), ASA classification, preoperative use of immunosuppressive drugs, preoperative radiation of the surgical site, indication for surgery, wound class, surgical procedure, duration of surgery and timing of perioperative intravenously administered antibiotic prophylaxis. Per-protocol analyses will be performed in addition to all the intention-to-treat analyses.

#### Interim analysis

One interim analysis will be performed when 50% of the participants completed the 30-day follow-up. The Z-statistic for the standardized treatment effect will be computed and compared with the O’Brien-Fleming type of boundaries for both efficacy and futility. Recruitment will stop if the Z statistic is larger than the efficacy boundary. The futility boundaries are non-binding and thus suggestive. The unblinded results of the interim analysis will be presented to an independent Data Safety and Monitoring Board (DSMB).

### Monitoring and safety

Independent Good Clinical Practice (GCP)-certified monitors from the UMC Utrecht will monitor the data collection and study progress. The frequency of these visits will be on a risk-based approach according to GCP guidelines.

Data entry will be verified using internal monitoring by a member of the coordinating study team. In addition, external monitoring will be performed by the independent monitors. During the monitoring visit, data entry will be verified on a random sample of the included patients by assessment of the source documents (e.g., electronic patient files, questionnaires or diaries). In the case of discrepancies or missing data, queries will be added to the questions that need revision or additional information.

#### Adverse events

Pre-OP consists of non-absorbable antibiotics. When taken orally, there is virtually no uptake of antibiotic components in the bloodstream. It is, therefore, expected that the occurrence of systemic side effects is negligible. More importantly, there is extensive experience with non-absorbable antibiotics applied as SDD on ICUs and severe and acute side effects that could apply to the current study have not been reported [27–30]. For this reason, we decided to refrain from an obligatory observation period after intake of the first dose of the study medication and patients can, therefore, take the medication at home. Nevertheless, patients will receive information about severe side effects of this study medication and are instructed to seek medical advice when they might suffer from severe acute side effects. The unblinding protocol can be freely assessed via our website, when deemed necessary. Late side effects, such as the development of antibiotic resistance, have not been described either [31–35]. Nevertheless, the occurrence of side effects and the development of antibiotic resistance will be monitored as secondary endpoints of this study.

All adverse events will be reported to the principle investigator. Serious adverse events (SAEs), serious adverse reactions (SARs) and suspected unexpected serious adverse reactions (SUSARs) will be reported to the sponsor within 24 h after notification of the event. For the SAEs, we decided to only report life-threatening infections with colistin- or tobramycin-resistant bacteria or *C. difficile* colitis within 24 h. Postoperative complications that are known to occur after colorectal surgery and are not directly related to the study medication will be reported to the ethical board in periodic line-listings and not within the 24-h time window.

#### Data Safety and Monitoring Board

A Data Safety and Monitoring Board (DSMB) is established to guard patient safety during the trial. The DSMB consists of four independent experts who will have an active role during the entire study period. The board will issue recommendations to continue or stop the study, based on the unblinded results of the interim analysis and on the list of SAEs, SARs and SUSARs.

## Discussion

Undergoing surgery is associated with an increased risk of surgical site infections, but also mortality. To overcome the issue of mortality being a competing risk with the development of deep SSIs, our primary outcome of interest, we decided to use a composite endpoint for our primary outcome. Thirty-day all-cause mortality and deep SSIs will also be analyzed separately as secondary endpoints.

The rationale behind our intervention is to decontaminate the digestive tract prior to surgery, where we hypothesize that by lowering the bacterial load in the colon and rectum during the surgical procedure, the risk of postoperative infections is reduced. In a previous study, a suspension of colistin sulphate, tobramycin and amphotericin B was used to reduce anastomotic leakage and postoperative infectious complications [24]. This suspension is identical to the suspension that is used as an antibiotic prophylaxis on Dutch ICUs, often referred to as SDD). In contrast with SDD, the solution that will be used in our study does not contain an antifungal component (amphotericin B or nystatin). Fungi are rarely identified as the causative pathogens of SSIs after surgical procedures on the lower gastrointestinal tract and it was, therefore, decided to omit the antifungal component [36]. Another difference between our study and the trial by Roos et al. is the timing of the treatment. In the previous trial, the intervention started 2 days prior to surgery and was continued during the postoperative period until normal bowel movements were observed. In our study, the intervention will only be given preoperatively and is discontinued on the night before the surgical procedure. We hypothesize that the majority of SSIs is caused by intraoperative rather than postoperative contamination of the surgical site and that the greatest reduction in SSIs will, therefore, be achieved by ensuring that the colonic bacterial load is at its lowermost at the time the surgical procedure takes place. The prophylaxis in our study is, therefore, restricted to the preoperative period. We also hypothesize that the treatment period of 3 days will be sufficient since we expect that the decontamination will least for several days. In our opinion, continuation of the prophylaxis in the postoperative period is not necessary as this could adversely increase the risk of developing antibiotic resistance or opportunistic infections.

To date, there is no consensus on the use of preoperative orally administered antibiotic prophylaxis prior to colorectal surgery in addition to perioperative intravenously administered antibiotic prophylaxis and without concurrent use of MBP. This impedes the formulation of clear-cut recommendations on its use in current infection control guidelines. This randomized, placebo-controlled, double-blind multicenter study aims to determine the efficacy of Pre-OP in addition to perioperative intravenously administered antimicrobial prophylaxis on the development of SSIs and other infectious and non-infectious complications. The proposed study design offers an optimal approach to adequately minimize bias and generate generalizable results in order to guide important evidence-based recommendations regarding infection control measures in colorectal surgery (Additional file 2).

### Trial status

The trial received ethical approval in September 2016. The recruitment of patients started in April 2017.

## Additional files

Additional file 1: Case Report Form. (DOCX 42 kb)

Additional file 2: SPIRIT 2013 Checklist used as a guideance for the development of the PreCaution study protocol. (DOC 120 kb)

## Abbreviations

CPE: Carbapenemase-producing *Enterobacteriaceae*
DSMB: Data Safety and Monitoring Board
eCRF: Electronic Case Report Form
ESBL: Extended-spectrum beta-lactamase
GCP: Good Clinical Practice
HRE: Highly resistant *Enterobacteriaceae*
ICU: Intensive care unit
MBP: Mechanical bowel preparation
Pre-OP: Preoperative orally administered antibiotic prophylaxis
SAE: Serious adverse event
SAR: Serious adverse reaction
SDD: Selective decontamination of the digestive tract
SSI: Surgical site infection
SUSAR: Suspected unexpected serious adverse reaction
