# Supplementary material for: Prevention of severe infectious complications after colorectal surgery using preoperative orally administered antibiotic prophylaxis (PreCaution): study protocol for a randomized controlled trial
Source: Trials. 2018 Jan 19;19:51. doi: 10.1186/s13063-018-2439-4 (PMC5775605; doi:10.1186/s13063-018-2439-4)
Supplement: Supplementary file 1 — Case Report Form. (DOCX 42 kb) [file 13063_2018_2439_MOESM1_ESM.docx]

|  | ***Inclusion*** | | |
| --- | --- | --- | --- |
|  | Date of inclusion | DD.MM.YYYY | |
|  | Site |  | |
|  | eCRF number | Will be generated automatically | |
|  | Medication number |  | |
|  | Month and year of birth | MM.YYYY | |
|  |  |  | |
|  | ***Exclusion criteria*** |  | |
|  | Please check the following exclusion criteria. NOTE: when one or more of the following questions is answered with YES, the patient is not eligible to participate | | |
|  | Patient is younger than 18 years of age | - No | - Yes |
|  | Patient is legally incapacitated | - No | - Yes |
|  | Patient is unable to take oral medication | - No | - Yes |
|  | Patient is pregnant or nursing | - No | - Yes |
|  | Patient has a known and documented allergy for colistin, tobramycin or other aminoglycoside antibiotics | - No | - Yes |
|  | Patient is diagnosed with myastenia gravis | - No | - Yes |
|  | Patient underwent abdominal surgery in the 30 days prior to inclusion | - No | - Yes |
|  | Patient has a stoma | - No | - Yes |
|  | Patient already participated in the PreCaution trial | - No | - Yes |
|  | Patient will undergo acute colorectal surgery | - No | - Yes |
|  |  | Inclusion | Exclusion |
|  | ***Preoperative work-up*** |  | |
|  | Did the patient receive the first quality of life questionnaire? | - No - Yes | |
|  | Rectal swab performed on the day of inclusion? | - No: not performed because: […..] - No: rectal swab performed on a different date: DD.MM.YYYY - Yes: rectal swab performed on the day of the inclusion | |

**Inclusion**

**Patient and surgery characteristics**

|  | ***Patient characteristics*** | | | | |
| --- | --- | --- | --- | --- | --- |
|  | Sex | | - Male - Female | | |
|  | ***The following questions apply to the hospital admission for the elective colorectal procedure*** | | | | |
|  | Date of admission | | DD.MM.YYYY | | |
|  | Date of surgery | | DD.MM.YYYY | | |
|  | Did de patient die during this hospital admission? | | - Yes - No | | |
|  | ***Pop-up question when answered with YES:*** date of death*?* | | DD.MM.YYYY | | |
|  | ***Pop-up question when answered with NO:*** date of discharge? | | - DD.MM.YYYY - Patient is still admitted | | |
|  | ASA classification | | - ASA 1 - ASA 2 - ASA 3 - ASA 4 - ASA 5 - Unknown | | |
|  | Length | | Range [30-230 cm] |  | |
|  | Weight | | Range [20-230 kg] |  |  |
|  | Preoperative serum albumin measured <1 week prior to surgery | | - [….] g/L - Unknown or not available | | |
|  | ***Comorbidities. When unknown, choose NO*** | | | | |
|  | ***Comorbidity*** |  | | |  |
|  | Myocardial infarction | - No - Yes | | |  |
|  | Heart failure | - No - Yes | | |  |
|  | Peripheral artery disease (including aortic aneurysms ≥ 6 cm) | - No - Yes | | |  |
|  | Cerebrovascular diseases | - No - Yes | | |  |
|  | Dementia | - No - Yes | | |  |
|  | Chronic pulmonary disease | - No - Yes | | |  |
|  | Chronic connective tissue disease | - No - Yes | | |  |
|  | Stomach ulcer | - No - Yes | | |  |
|  | Mild liver disease | - No - Yes | | |  |
|  | Moderate to severe liver disease | - No - Yes | | |  |
|  | Diabetes mellitus without complications | - No - Yes | | |  |
|  | Diabetes mellitus with complications | - No - Yes | | |  |
|  | Paraplegia | - No - Yes | | |  |
|  | Moderate to severe renal insufficiency | - No - Yes | | |  |
|  | Solid malignancy without metastasis | - No - Yes | | |  |
|  | Solid malignancy with metastasis | - No - Yes | | |  |
|  | Leukemia | - No - Yes | | |  |
|  | Lymphoma | - No - Yes | | |  |
|  | Immunodeficiency: | | | |  |
|  | - AIDS - Neutropenia (<200 neurophils <1 week prior to surgery) - Recipient of donor bone marrow or donor organ(s) - Splenectomy - Primary immunodeficiency syndrome | - No - Yes - No - Yes - No - Yes - No - Yes - No - Yes | | |  |

|  | ***Treatment prior to surgery*** | | |
| --- | --- | --- | --- |
|  | Use of immunosuppressive therapy in the 30 days prior to surgery   - Chemotherapy - Corticosteroids, min. of 14 consecutive days in the past 30 days, 0.5 mg/kg/day - Other immunosuppressives | | - Nee - Ja - Ja - Nee - See drop down menu |
|  | Radiotherapy surrounding the surgical site within the 30 days prior to surgery | | - No - Yes - Unknown |
|  | Abdominal surgery in the past year | | - No - Yes - Unknown |
|  | Mechanical bowel preparation prior to surgery | | - Oral preparation (e.g. PEG) - Enema - No preparation |
|  | ***Compliance to the study medication*** | | |
|  | Leftovers of study medication collected? | | - No - Yes |
|  | ***Pop-up question when answered with YES: weight of the leftovers of study medication*** | | [……] gram |
|  | Medication diary collected? | | - No - Yes |
|  | ***Pop-up question when answered with YES: which complaints are mentioned in the diary?*** | | |
|  | **Complaint (drop down menu)** | **Severity / times per day (drop down menu)** | **How many days (drop down menu)** |
|  | Diarrhea | 1  2  3  4  5  6  7  8  9  10  >10 | 1  2  3 |
|  | Stomach ache |  |  |
|  | Nausea |  |  |
|  | Other: …….. |  |  |
|  | Other: …….. |  |  |
|  | No complaints mentioned by the patient | | |
|  | *Open text to fill in new / other complaints* | | |
|  | *Warning: when a patient experienced side effects, do not forget to fill in the adverse event form (separate form in eCRF)* | | |

|  | ***Surgery characteristics*** | |
| --- | --- | --- |
|  | Indication for surgery | - Colorectal cancer - Inflammatory bowel disease - Polyposis coli - Diverticulitis - Non resectable polyp - Other: [open text] |
|  | Wound classification | - Wound class 2: clean/contaminated - Wound class 3: contaminated - Wound class 4: dirty - Unknown |
|  | Type of procedure | - Right sided hemicolectomy - Left sided hemicolectomy - Sigmoidectomy - (low) anterior resection - Rectum amputation - Subtotal colectomy - Total proctocolectomy - Other: [open tekst] |
|  | Surgery technique | - Laparotomy (open procedure) - Laparoscopy without conversion to laparotomy - Laparoscopy with conversion to laparotomy - Robotic surgery - Unknown |
|  | Stoma created during surgery? | - Yes - No - Unknown |
|  | Time first incision | - hh:mm - Unknown |
|  | Time closure of the wound | - hh:mm - Unknown |
|  | Implants of non-human tissue | - No - Yes |
|  | ***Pop-up question when answered with YES: what kind of tissue was implanted?*** | - Plastic - Biologic - Unknown |
|  | Blood transfusion during surgery? | - No or not documented - Yes |
|  | Body temperature during surgery | |
|  | First measured temperature after the first incision | - […….] Celsius - Unknown |
|  | Last measured temperature before closure of the wound | - [………] Celsius - Unknown |
|  | Other types of surgery performed during this procedure (e.g. resection of liver metastases. Creation of stoma does not count as second procedure) | - No - Yes |
|  | ***Perioperative intravenous antibiotic prophylaxis*** | |
|  | Perioperative prophylaxis administered? | - No - Yes - Unknown |
|  | ***Pop-up questions when answered with YES :*** |  |
|  | Time of first dose of antibiotics | - hh:mm - Unknown |
|  | Second dose of antibiotics administered during the procedure? | 2^e^ dosis: hh:mm  *+ add extra doses if applicable* |
|  | Select antibiotic(s) that is/are administered | - Cefazolin (Kefzol) - Metronidazole (Flagyl) - Cefoxitin (Mefoxin) - Amoxicillin with clavulanic acid (Augmentin) - Amoxicillin - Other,: open text |

**Endpoints 30 days after surgery**

| **Surgical site infection** | | | | |
| --- | --- | --- | --- | --- |
|  | Surgical reintervention within 30 days? | - No - Yes, date DD.MM.YYYY | | |
|  | ***Pop-up questions when answered with YES:*** *indication for reintervention?* | - (suspicion of) bleeding - (suspicion of) infection - (suspicion of) anastomotic leakage - Other: [open text] | | |
|  | Microbiological culture taken during this procedure? | - No - Yes | | |
|  | ***Pop-up question when answered with YES:*** ***date of culture and result***  Note: if more than one culture is taken, fill in the date of the first positive culture. If all cultures were negative, fill in date of first negative culture. | - Date DD.MM.YYYY culture was negative - Date DD.MM.YYYY culture was positive | | |
|  | ***Pop-up question when answered with YES:*** *leukocytes present in gram staining?* | - None or a few - Several to many - Unknown | | |
|  | Culture of wound or pus performed on another moment than the surgical reintervention | - No - Yes | | |
|  | ***Pop-up question when answered with YES:*** *Result of this culture*  Note: if more than one culture is taken, fill in the date of the first positive culture. If all cultures were negative, fill in date of first negative culture. | - Date DD.MM.YYYY, culture was negative - Date DD.MM.YYYY, culture was positive | | |
|  | Incision surgically opened? | - No - Yes, date: DD.MM.YYYY | | |
|  | Wound dehistence? | - No - Yes, date: DD.MM.YYYY | | |
|  | Pus or purulent drainage from wound? | - No - Yes, date: DD.MM.YYYY | | |
|  | Pus or purulent drainage from wound drain? | - No - Yes, date: DD.MM.YYYY | | |
|  | Abdominal abscess? | - No - Yes, date: DD.MM.YYYY | | |
|  | One of more signs or symptoms mentioned? Multiple answers may apply | - Localized pain or tenderness surrounding the incision - Local swelling surrounding the incision - Local redness surrounding the incision - fever (>38 degrees Celsius) - heat (skin) - No signs or symptoms of infection | | |
|  | ***Pop-up question when 1 or more signs / symptoms are present:***  *Date on which these signs are mentioned for the first time* | **DD.MM.YYYY** | | |
|  | Antibiotics used during hospital admission within 30 days after surgery?  Note: started at least 24 hours after surgery.  Note: also when patient is readmitted within 30 days after surgery | - No - Yes | | |
| **A** | ***Pop-up question when answered with YES*** *:* *What was the indication for antibiotic treatment?* | - wound infection suspected - other infection suspected - Unknown | | |
|  | ***Pop-up question when answered with YES*** : **Please indicate the antibiotics that were used, including the date of start and end of treatment** | | | |
| **B** | Antibiotic list | | Start date | Stop date |
|  | Drop down menu | | DD.MM.YYYY | DD.MM.YYYY |
|  | In case of switch of antibiotic therapy or start of new antibiotics but with same indication for treatment: add new line B with new dates in CRF. In case of a new indication for treatment, add new line A | | | |
|  | Diagnosis of superficial surgical site infection by surgeon of attending physician? | - No - Yes, date: DD.MM.YYYY | | |
|  | Diagnosis of deep surgical site infection by surgeon of attending physician?? | - No - Yes, date: DD.MM.YYYY | | |
|  | ***Open text field for notes*** | | | |

**Anastomotic leakage**

|  | One or more of the following signs / symptoms mentioned?  Multiple answers may apply | - Abdominal or pelvic pain - Fever >38 degrees Celcius - Signs of peritonitis - Pus of fecal discharge from wound or wound drain - None of the above |
| --- | --- | --- |
|  | ***Pop-up question when one or more answers is/are selected:*** *fill in the* *date on which this / these sign(s) are mentioned for the first time* | - DD.MM.YYYY |
|  | Additional blood tests performed? | - No - Yes |
|  | ***Pop-up question when answered with YES:*** *findings?* | - Increased CRP - Leukocytosis - No increased CRP and no leukocytosis |
|  | Additional radiologic tests performed? | - No - Yes |
|  | ***Pop-up question when answered with YES****: findings?* | - No abnormalities or findings not suspected for anastomotic leakage - Suspected for anastomotic leakage |
|  |  |  |

**30-day microbiological endpoints will be collected at the end of the study**

**30 day mortality**

|  | Did someone contact patient or his/her family? | - Yes - No |
| --- | --- | --- |
|  | Did the patient die within 30 days after surgery | - Yes - No |
|  | ***Pop-up question when answered with YES***, *date of death* | DD.MM.YYYY |
|  | ***Pop-up question when answered with NO,*** *rectal swab sent to patient?* | - No, please sent the swab including the instructions - Yes |

**Evaluation 6 months after surgery**

**6-month mortality**

|  | Did someone contact patient or his/her family? | - Yes - No |
| --- | --- | --- |
|  | Did the patient die within 6 months after surgery? | - Yes - No |
|  | ***Pop-up question when answered with YES***, *date of death* | DD.MM.YYYY |
|  | ***Pop-up question when answered with NO*** *quality of life questionnaire sent to patient?* | - No, please sent the questionnaire - Yes |

**Readmissions and length of stay**

|  | Was the patient readmitted to the hospital within 6 months after the elective surgical procedure | | | - No - Yes |
| --- | --- | --- | --- | --- |
|  | ***Pop-up question when answered with YES***: *please fill in the date of admission and date of discharge of each hospital admission. This concerns admission to the hospital in general.* | | | |
|  | **Admission** | **Date of admission** | | **Date of discharge** |
|  | Admission 1 | DD.MM.YYYY | | DD.MM.YYYY |
|  | + For every hospital admission, please create a new row in the CRF | | | |
|  | Was the patient admitted to the ICU within 6 months after the elective surgical procedure? | | | - No - Yes |
|  | ***Pop-up question when answered with YES****: please fill in the date of admission and date of discharge of each ICU admission* | | | |
|  | **ICU admission** | | **ICU date of admission** | **IC date of discharge** |
|  | ICU admission 1 | | DD.MM.YYYY | DD.MM.YYYY |
|  | + For every ICU admission, please create a new row in the CRF | | | |
|  | | | | |

**Quality of life questionnaire**

Paper version sent to the patient (RAND-36)

CRF also includes an adverse event form where complaints and side effects are documented. SAE and SUSARs will be documented on paper forms.
